# Anatomical Distribution of Ochronotic Pigment in Alkaptonuric Mice is Associated with Calcified Cartilage Chondrocytes at Osteochondral Interfaces

**DOI:** 10.1007/s00223-020-00764-6

**Published:** 2020-10-14

**Authors:** Juliette H. Hughes, Craig M. Keenan, Hazel Sutherland, Henry R. Edwards, Peter J. M. Wilson, Lakshminarayan R. Ranganath, Jonathan C. Jarvis, George Bou-Gharios, James A. Gallagher

**Affiliations:** 1 Department of Musculoskeletal & Ageing Science, Institute of Life Course and Medical Science, University of Liverpool, William Henry Duncan Building, 6 West Derby Street, Liverpool, L7 8TX UK; 2 School of Biological and Environmental Sciences, Faculty of Science, Liverpool John Moores University, Liverpool, L3 3AF UK; 3 Department of Clinical Biochemistry and Metabolic Medicine, Royal Liverpool University Hospital, Liverpool, L7 8XP UK; 4 School of Sport and Exercise Science, Faculty of Science, Liverpool John Moores University, Liverpool, L3 3AF UK

**Keywords:** Alkaptonuria, Ochronosis, Calcified cartilage, Chondrocyte, Extracellular matrix, Mouse model

## Abstract

Alkaptonuria (AKU) is characterised by increased circulating homogentisic acid and deposition of ochronotic pigment in collagen-rich connective tissues (ochronosis), stiffening the tissue. This process over many years leads to a painful and severe osteoarthropathy, particularly affecting the cartilage of the spine and large weight bearing joints. Evidence in human AKU tissue suggests that pigment binds to collagen. The exposed collagen hypothesis suggests that collagen is initially protected from ochronosis, and that ageing and mechanical loading causes loss of protective molecules, allowing pigment binding. Schmorl’s staining has previously demonstrated knee joint ochronosis in AKU mice. This study documents more comprehensively the anatomical distribution of ochronosis in two AKU mouse models (BALB/c *Hgd*^*−/−*^, *Hgd tm1a*^*−/−*^), using Schmorl’s staining. Progression of knee joint pigmentation with age in the two AKU mouse models was comparable. Within the knee, hip, shoulder, elbow and wrist joints, pigmentation was associated with chondrons of calcified cartilage. Pigmented chondrons were identified in calcified endplates of intervertebral discs and the calcified knee joint meniscus, suggesting that calcified tissues are more susceptible to pigmentation. There were significantly more pigmented chondrons in lumbar versus tail intervertebral disc endplates (*p* = 0.002) and clusters of pigmented chondrons were observed at the insertions of ligaments and tendons. These observations suggest that loading/strain may be associated with increased pigmentation but needs further experimental investigation. The calcified cartilage may be the first joint tissue to acquire matrix damage, most likely to collagen, through normal ageing and physiological loading, as it is the first to become susceptible to pigmentation.

## Introduction

Alkaptonuria (AKU; OMIM #203500) is a rare inherited metabolic bone disease caused by deficiency of homogentisate 1,2-dioxygenase (HGD; EC 1.13.11.5) [1]. HGD catalyses the breakdown of homogentisic acid (HGA) to 4-maleylacetoacetic acid in the tyrosine catabolic pathway, and therefore HGD deficiency causes increased HGA in the urine, blood and tissues [2, 3]. Most HGA is excreted but over time some is deposited as a dark pigment in connective tissues, primarily the cartilage of loaded joints. This formation of pigment, probably through oxidation of HGA, is termed ochronosis and causes tissues to become dark in appearance and brittle [2, 4, 5]. In cartilage this leads to the early-onset and severe debilitating osteoarthropathy that characterises AKU.

Ochronotic pigment has been identified in connective tissues throughout the body of AKU patients at surgery or autopsy, but articular cartilage appears to be the most susceptible tissue. Ochronosis has been found in the articular cartilage of large weight bearing joints (knee, hip, shoulder, elbow), and in many other hyaline cartilages, including the costal cartilages, larynx, trachea and bronchi, sternoclavicular and sacroiliac joints [3, 6–9]. Pigmentation of fibrocartilaginous tissue such as the annulus fibrosus of intervertebral discs (IVDs), pubic symphysis [7, 9] and knee joint meniscus [10, 11], and the elastic cartilage of the external ear and epiglottis [3, 9] has also been observed. Non-cartilaginous tissues such as ligaments, tendons, synovial membrane, periosteum (although not the bone itself), perichondrium, sclera and conjunctiva of the eye, skin and dura of the brain are also reported to become pigmented [3, 7, 9, 12–14]. Within the cardiovascular system, pigmentation has been identified in heart valves, chordae tendineae and the roots and walls of major arteries such as the aorta, where pigmentation was present in the intima and adventitia of the arterial wall [6, 7, 9, 13].

**Fig. 1:**
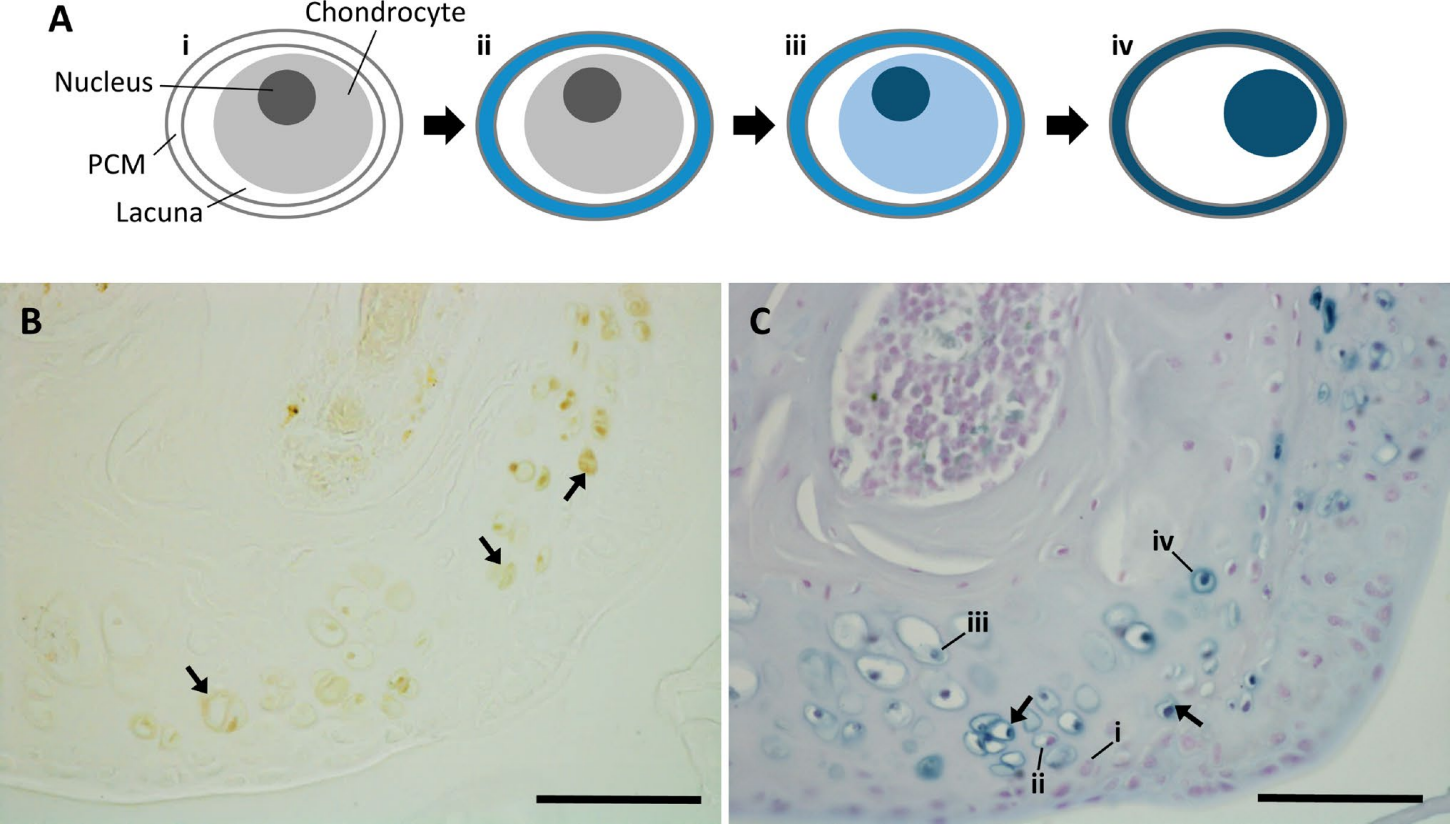
Schmorl’s staining of ochronotic pigment in articular calcified cartilage chondrons. **a** A diagrammatic representation of pigmentation progression from a healthy, unpigmented chondron (i) to a heavily pigmented, pyknotic chondron (iv). The pericellular matrix (PCM) pigments first (chondron ii), followed by intracellular pigment (chondron iii) which both get more intense over time (chondron iv). **b** Unstained ochronotic pigment (yellow-brown colour) in the medial femoral condyle of the knee joint (arrowed). **c** A near serial section of ochronotic pigment stained with Schmorl’s staining (dark blue colour), with arrows showing examples of pigmented chondrons within the articular calcified cartilage. Chondrons at the stages shown in (**a**) are identified in (**c**). Tissue from 65.7-week-old (F) BALB/c *Hgd*^*−/−*^ mouse. Scale bar = 50 µm

Although the exact mechanism of pigment deposition/formation is currently unknown, the pattern and progression of this pigmentation within cartilage has been determined by histological analysis of AKU cartilage from both humans and mice. Human AKU cartilage samples harvested during joint replacement surgery showed anatomical gradients of pigmentation from lightly pigmented to heavily pigmented areas and indicated that initial pigmentation was associated with individual chondrocytes located in the articular calcified cartilage (ACC) [5]. Further examination in mice showed that pigment was first observed in the pericellular matrix of chondrons within the ACC surrounding the cell like a “halo” with no cellular staining, and then advanced to the cellular compartment [15] as depicted in Fig. 1a. Although pigmentation was limited to the ACC in mouse knee joints, in human AKU cartilage pigmentation progressed from the ACC to the deep and middle hyaline cartilage zones observed as a blanket pigmentation of the extracellular matrix (ECM), and then advanced to the superficial hyaline cartilage layer where it appears to spread much faster [5]. In the most heavily pigmented samples, a complete absence of the ACC and subchondral bone (SCB) was observed. It is hypothesised that this phenomenon is due to stress shielding by the heavily pigmented and therefore stiffer hyaline cartilage zones above, leading to osteoclastic resorption in the unloaded ACC and SCB [5].

The structure of ochronotic pigment, and the mechanism of its deposition/formation remains unknown, as recently discussed in a review by Ranganath et al*.* [16]; it is not known whether ochronosis occurs by initial binding of HGA itself, or an oxidised intermediate, or as ochronotic pigment [16]. Ochronotic pigment is deposited in connective tissues, most of which contain collagen as a key component of the ECM. In human tissue, it has been shown that ochronotic pigment has an affinity for fibrillar collagen surrounded by glycosaminoglycan-rich ground substance, particularly in hyaline cartilage [13]. Transmission electron microscopy analysis of the ligamentous capsule of an AKU hip joint revealed extracellular granular pigmentation associated with collagen fibres, with the pigmentation showing a periodic banding pattern on individual collagen fibres [17]. Taylor et al*.* suggested that the ECM is normally resistant to pigmentation and becomes susceptible after biochemical or mechanical damage [5]. They also demonstrated that pigmented cartilage is much stiffer than non-pigmented cartilage, and suggested that focal pigmentation of individual chondrocytes and their associated collagen is likely to make the ECM prone to more damage through normal loading, leading to further damage in an inevitable downward spiral. This led to the exposed collagen hypothesis as the small granule pigmentation observed on collagen fibres appeared similar to the pattern of proteoglycan binding, leading to the theory that proteoglycan and glycosaminoglycan loss exposes collagen fibrils, allowing initial HGA/pigment binding that acts as a nucleation point for pigment deposition [18]. Solid state NMR studies have shown nanoscale collagen disorder and proteoglycan loss in AKU cartilage, supporting the theory that loss of proteoglycans and other molecules, due to damage, may allow HGA binding [19].

HGA is a reducing agent and can be rapidly oxidised to benzoquinone acetic acid under alkaline conditions, turning solutions and tissues black [20]. A modified version of Schmorl’s stain, usually used to identify melanin pigments, has been used both in cells incubated with HGA (in vitro) [4] and in human AKU tissue sections [21, 22] to enhance ochronotic pigment from a yellow-brown to an intense blue-green colour, by the reduction of ferricyanide to ferrocyanide by ochronotic pigment [23]. A recent analysis of human AKU pigmented cartilage using NMR indicated the presence of hydroquinones that are known for their redox activity, and also identified radicals via EPR (electron paramagnetic resonance) spectroscopy of synthetic pigment [24]. Changes in glycine signals were observed in the pigmented cartilage samples, most likely indicating intrastrand disruption of the collagen triple helix due to a disruption in hydrogen bonds involving glycine, that probably changes the mechanical properties of the cartilage [24]. The authors propose that formation of glycyl radicals, enabled by HGA radical formation, can disrupt hydrogen bonding within the collagen triple helix and oxidise nearby HGA molecules, amplifying the process of HGA oxidation, increasing pigment formation and leading to further collagen damage.

Studying human ochronosis within cartilage has revealed important information regarding the pattern and location of pigmentation. However, the study of diseased joints is limited to samples with advanced disease, which are only obtainable at surgery or autopsy. AKU mice (here denoted BALB/c *Hgd*^*−/−*^) were first identified at the Pasteur Institute after ENU (ethylnitrosourea) mutagenesis [25, 26]. Early reports showed that the mice displayed the biochemical phenotype of human AKU but with no ochronosis identified macroscopically or histologically. Schmorl’s staining of tissue sections was subsequently used to identify ochronosis in the ACC of the knee joint associated with chondrons as described above, with no pigment noted in the bone or non-calcified hyaline cartilage [15, 27]. Pigmented chondrons in these mice were only ever observed in the ACC and in contrast to advanced ochronosis in weight bearing human joints, no inter-territorial blanket pigmentation was observed, even at 71 weeks of age [15]. A new targeted mouse model of AKU (*Hgd tm1a*^*−/−*^; C57BL/6) also recently reported knee joint ochronosis with pigmentation confined to chondrons of ACC in mice aged 9, 26 and 40 weeks [28].

These previous reports of ochronosis in AKU mouse models were restricted to qualitative observations of the knee joint. Here, the tissue distribution of ochronotic pigment within a variety of AKU mouse tissues was studied to determine the tissues/cell types susceptible to pigmentation. Identifying the first sites of pigment deposition, which is not possible in human AKU tissues, along with potential factors affecting its severity, provides direction for future work aiming to identify not only the structure of ochronotic pigment and its mechanism/location of deposition but also to provide targets for its prevention or clearance from affected tissue. Joints other than the knee were therefore investigated, including both large and small joints, in addition to the eye, ear, laryngeal cartilage and the heart, which are all documented sites of ochronosis in human AKU. Studying the pigmentation of chondrons in areas of greater compressive loading, such as the lumbar vertebrae compared to the tail vertebrae, and areas of tensile strain such as ligament entheses highlights a potential relationship between physiological loading and increased susceptibility to ochronotic pigmentation. Additionally, our observations of ochronotic pigment distribution in AKU mice may reveal the order of load- and age-induced changes that occur during normal, healthy ageing in articular cartilage.

## Materials and Methods

### Mice

Two AKU mouse models, BALB/c *Hgd*^*−/−*^ [15] and *Hgd tm1a*^*−/−*^ [28], were used to explore ochronosis. Tissues were collected from male (M) and female (F) mice, ranging from 7 to 66 weeks of age (see figure legends). All mice were housed, maintained and experimental procedures carried out within the University of Liverpool Biological Services Unit in specific pathogen-free conditions, under project licence 70/9047, in accordance with UK Home Office guidelines, under the Animals (Scientific Procedures) Act 1986. Food and water were available ad libitum. Mice were culled by Schedule 1 approved methods and tissues harvested without delay.

### Tissue preparation

Large joints including the knee, hip, elbow, shoulder and vertebral column and small joints of the wrist that included the articulations of the carpal bones with the metacarpals, ulnar and radius were collected to investigate pigmentation of joints. Other tissues reported to be pigmented in human AKU were also collected and included the ear, eye, tracheal and laryngeal cartilages and the heart. Dissected tissues were fixed overnight in 10% formalin. Hard tissues were then decalcified using 10% EDTA (ethylenediaminetetraacetic acid). Paraffin wax sections (5–6 µm) were stained with a modified Schmorl’s stain to enhance ochronotic pigment from a yellow-brown to a dark blue-green colour, which has previously been used in AKU mouse tissue sections to identify pigment [15, 27–29]. Briefly, tissues were deparaffinised, rehydrated, placed in Schmorl’s stain (1% ferric chloride, 1% potassium ferricyanide in distilled water) for up to 15 min, immersed in 1% acetic acid, and then counterstained with nuclear fast red. After washing, the stained sections were dehydrated, cleared and mounted with DPX. Sections were imaged using light microscopy, with a Nikon Eclipse Ci microscope (Nikon, Japan) equipped with a Ds-Fi2 camera and NIS Elements Br software (version 4.13.04).

### Quantification of Pigmented Chondrons

To score the severity of ochronosis within the tibio-femoral joint, the total number of pigmented chondrons (the chondrocyte and its surrounding pericellular matrix) in a full coronal section that has been Schmorl’s stained were counted using the method previously described by Keenan et al*.* [29]. Pigmented chondrons in the ACC were counted in all 4 joint quadrants (medial and lateral femur, medial and lateral tibia), including chondrons located in the entheses of the femur. This ochronosis scoring system was then adapted to the vertebral column; pigmented chondrons were counted in both the cranial and caudal endplates either side of an intervertebral disc (IVD) stained with Schmorl’s stain, across 5 coronal sections spaced approximately 15 µm apart. The 2nd lumbar (L2) IVD and an IVD from the base of the tail were examined. Scoring was carried out blind by two observers.

## Results

**Fig. 2:**
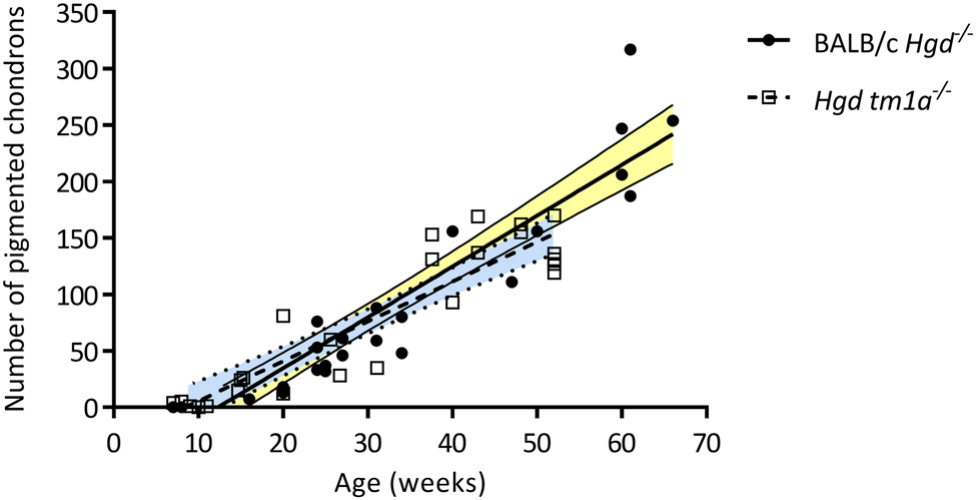
Quantification of pigmented chondrons with age in BALB/c *Hgd*^*−/−*^ and *Hgd tm1a*^*−/−*^ mice. The number of pigmented chondrons was quantified in a representative knee joint section stained with Schmorl’s stain from mice of various ages from both the BALB/c *Hgd*^*−/−*^ (*n* = 28; 16 female 7–66 weeks old, 12 male 8–47 weeks old) and *Hgd tm1a*^*−/−*^ (*n* = 25; 8 female 8–48 weeks old, 17 male 7–52 weeks old) mouse models. Progression of pigmentation was similar in both models, with the number of pigmented chondrons increasing with age. Lines show linear regression with 95% confidence bands; yellow shading represents BALB/c *Hgd*^*−/−*^*,* blue shading represents *Hgd tm1a*^*−/−*^

Ochronotic pigment was visible without staining in chondrons of the ACC in the knee joint of a 65.7-week-old BALB/c *Hgd*^*−/−*^ AKU mouse, which can be seen as both intracellular and extracellular yellow-brown pigment (Fig. 1b, arrowed). However, staining of a near serial section with Schmorl’s allowed for much easier identification of pigmented chondrocytes enhancing the colour of the ochronotic pigment to a dark blue-green colour (Fig. 1c, arrowed). Examples of each of the chondron pigmentation stages illustrated in Fig. 1a are identified in Fig. 1c, with pigmentation beginning in the pericellular matrix, and then advancing to the chondrocyte itself, as both cytoplasmic and nuclear pigmentation, with an increase in the intensity of staining also observed. The lifetime progression of the number of pigmented chondrons within the knee joint of the BALB/c *Hgd*^*−/−*^ mouse model has previously been reported [15, 29], and is compared to the newer *Hgd tm1a*^*−/−*^ model in Fig. 2, with both the BALB/c *Hgd*^*−/−*^ and *Hgd tm1a*^*−/−*^ models showing a remarkably similar linear increase in the number of pigmented chondrons with increasing age in the knee joint (BALB/c *Hgd*^*−/−*^* R*^2^ = 0.886, *p* < 0.001; *Hgd tm1a*^*−/−*^* R*^2^ = 0.840, *p* < 0.001), with overlapping 95% confidence bands.

**Fig. 3:**
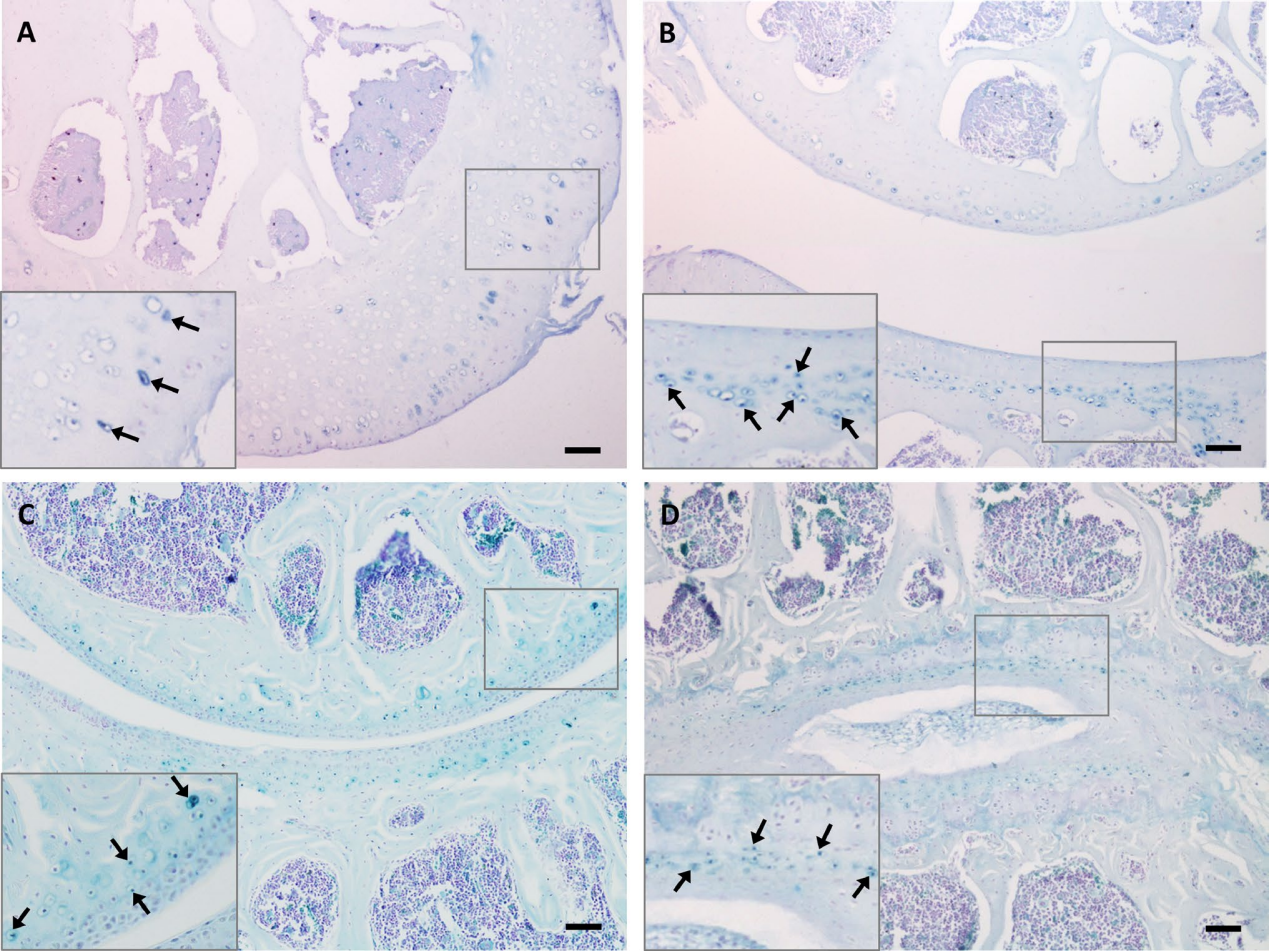
Schmorl’s staining showing ochronotic pigmentation in various joints. **a** Pigmented chondrons (arrowed) were visible in the femoral head of the hip joint, **b** the lateral epicondyle of the humerus and the radius of the elbow joint, and **c** the humeral head and glenoid fossa of the shoulder joint. **d** Pigmented chondrons (arrowed) were observed in the endplates of the vertebral bodies, either side of the intervertebral disc, but not within the disc itself. A + B from 65.7-week-old (F) BALB/c *Hgd*^*−/−*^. C from 52-week-old (F) *Hgd tm1a*^*−/−*^. D from 52-week-old (M) *Hgd tm1a*^*−/−*^. Scale bar = 50 µm

**Fig. 4:**
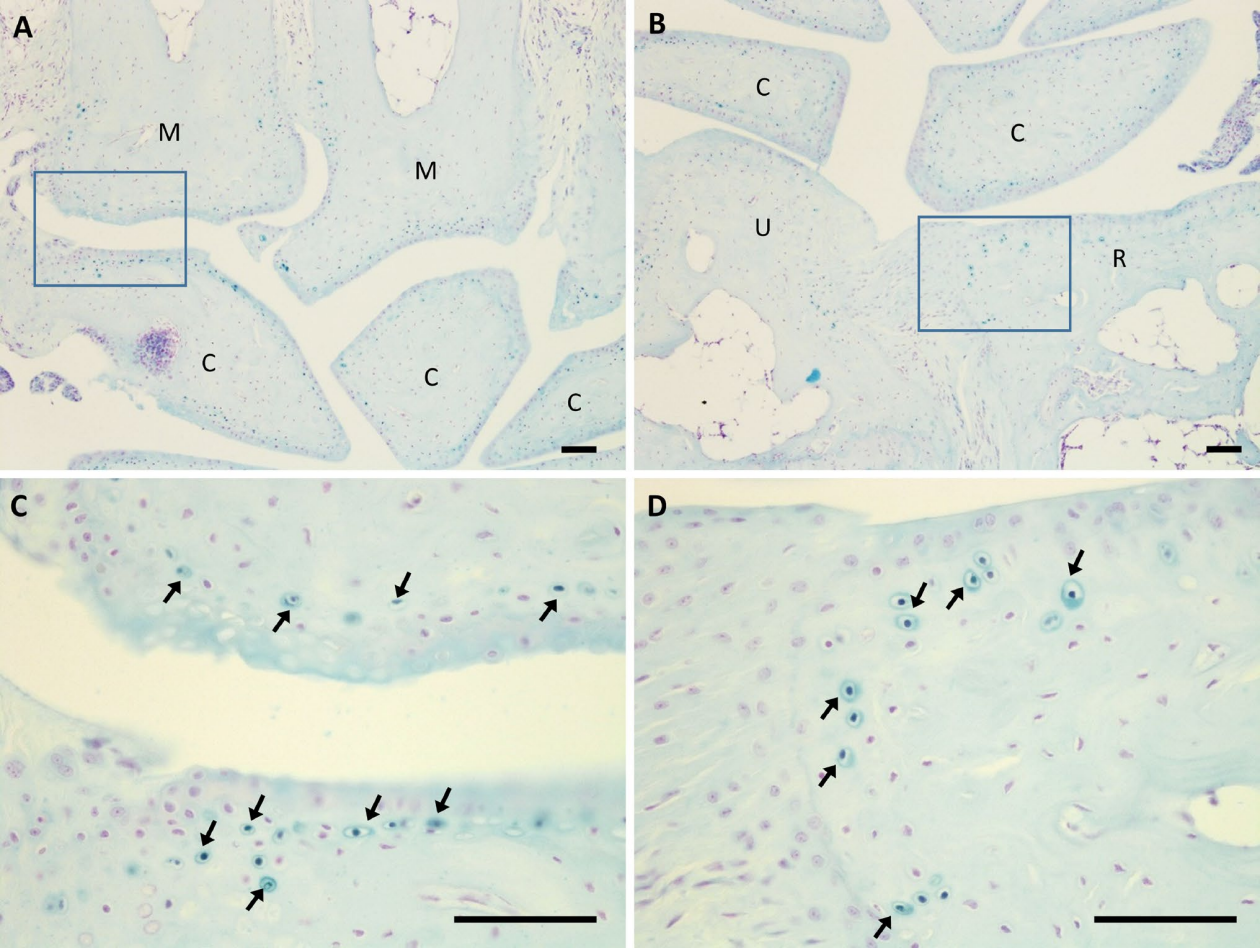
Schmorl’s staining showing ochronotic pigmentation in small forelimb wrist joints. Pigmented chondrons were observed in the calcified articular cartilage of all joint articulations within the wrist. **a** The articulations of the proximal metacarpals with the carpal bones and **b** the articulations of the distal radius and ulnar with carpal bones. **c** and **d** Higher magnification images of the boxes shown in **a** and **b**, respectively; pigmented chondrons (arrowed) are observed in the calcified articular cartilage. Also observed in both A + B are inter-carpal joints. Tissue from 52-week-old *Hgd tm1a*^*−/−*^ (M) mouse. M = metacarpal. C = carpal. R = radius. U = ulnar. Scale bar = 50 µm

Previously, ochronotic pigmentation in AKU mice has only been reported in the ACC of the knee joint. Here we show that histological analysis of the elbow, hip and shoulder joints, from both BALB/c *Hgd*^*−/−*^ and *Hgd tm1a*^*−/−*^ mice, identified pigmented chondrons within the ACC (Fig. 3a–c). Also investigated were the smaller joints of the forelimb paw (Fig. 4). Within the wrist, pigmented chondrons were observed within the calcified cartilage of all articulations examined, which included carpometacarpal (Fig. 4c), inter-carpal (Fig. 4a, b) and carporadial (Fig. 4d) and carpoulnar joints (Fig. 4b). No pigmentation was observed in the non-calcified hyaline cartilage, bone or periosteum of all investigated joints. No pigmentation was observed in the ligaments or tendons of all joints examined; however, pigmented chondrons were observed within the calcified fibrocartilaginous entheses at the insertion of the tendon/ligament at the bone/cartilage interface, see Fig. 7a–c for examples. Within the vertebral column, pigmentation was only associated with chondrons located in the calcified endplates of the vertebral bodies, adjacent to the growth plate, and not within the intervertebral disc (IVD) itself (Fig. 3d). The nucleus pulposus at the centre of the IVD in Fig. 3d has stained a dark blue colour, which is more apparent in the 2nd lumbar IVD shown in Fig. 6a. Schmorl’s staining of non-AKU, 52-week-old *Hgd tm1a*^*−/*+^ (female) vertebrae and IVDs, however, also results in blue staining of the nucleus pulposus as observed in *Hgd tm1a*^*−/−*^ IVDs; therefore it is not considered to be ochronotic pigmentation. Schmorl’s staining of joint tissue from non-AKU, 52-week-old Hgd tm1a^*−/*+^ (female) revealed no pigmentation in all joints assessed, and included the knee, hip, shoulder, elbow, wrist and the vertebral column.

**Fig. 5:**
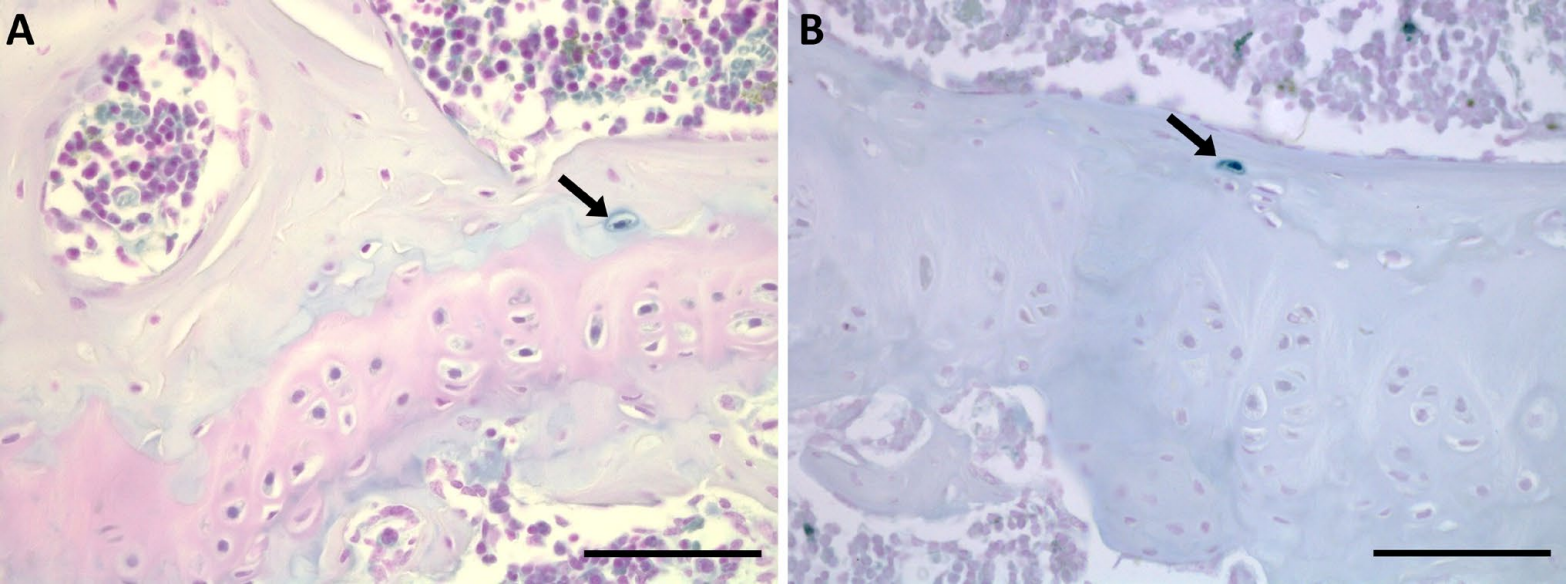
Detection of pigmented chondrons associated with the growth plate. A single pigmented chondron (see arrow) was observed in the resting zone of the tibial growth plate of a 40-week-old male *Hgd tm1a*^*−/−*^ mouse (**a**) and 60-week-old female BALB/c *Hgd*^*−/−*^ mouse (**b**). Scale bar = 50 µm

The knee joint sections stained with Schmorl’s stain and used for counting the number of pigmented chondrons with age in Fig. 2 were assessed (*Hgd tm1a*^*−/−*^* n* = 25; BALB/c *Hgd*^*−/−*^* n* = 28) to determine if there was any pigmentation associated with the tibial growth plate. In the majority of the mice, no pigmented chondrons were identified in the tibial growth plate. In two *Hgd tm1a*^*−/−*^ mice, aged 38- and 40 weeks old, and 3 BALB/c *Hgd*^*−/−*^ mice aged 60- (*n* = 2) and 66 weeks old (*n* = 1), a single pigmented chondron, however, was observed in the growth plate region. An example of a pigmented chondron associated with the tibial growth plate from both a *Hgd tm1a*^*−/−*^ and BALB/c *Hgd*^*−/−*^ mouse is shown in Fig. 5. In all 5 of mice, the single pigmented chondron identified was situated at the superior aspect of the growth plate in the resting zone.

**Fig. 6:**
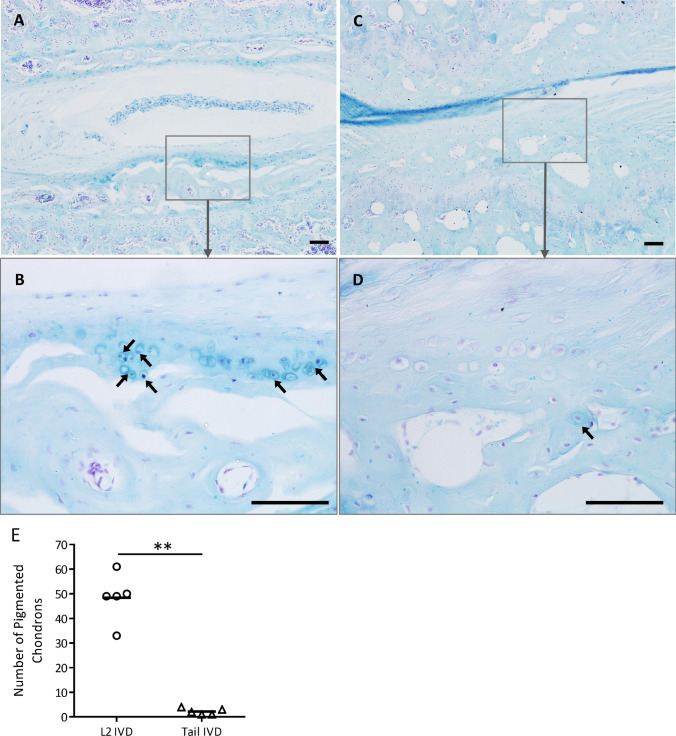
Comparison of pigmentation within the endplates of tail and lumbar IVDs. **a** + **b** Pigmented chondrons (arrowed) were observed in the vertebral endplates of the L2 IVD from the lumbar spine. **c** + **d** A small number of pigmented chondrons (arrowed) were present in the vertebral endplates of an IVD from the base of the tail. **e** Difference in the number of pigmented chondrons within the endplates of the L2 and tail IVD; 5 sections were scored, taken every 3rd slide. IVD = intervertebral disc. Two-tailed, unpaired *t*-test; *p* = 0.002. Significance: *p* < 0.05*, *p* < 0.01**, *p* < 0.001***. Tissue from 52-week-old (M) *Hgd tm1a*^*−/−*^. Scale bar = 50 µm

**Fig. 7:**
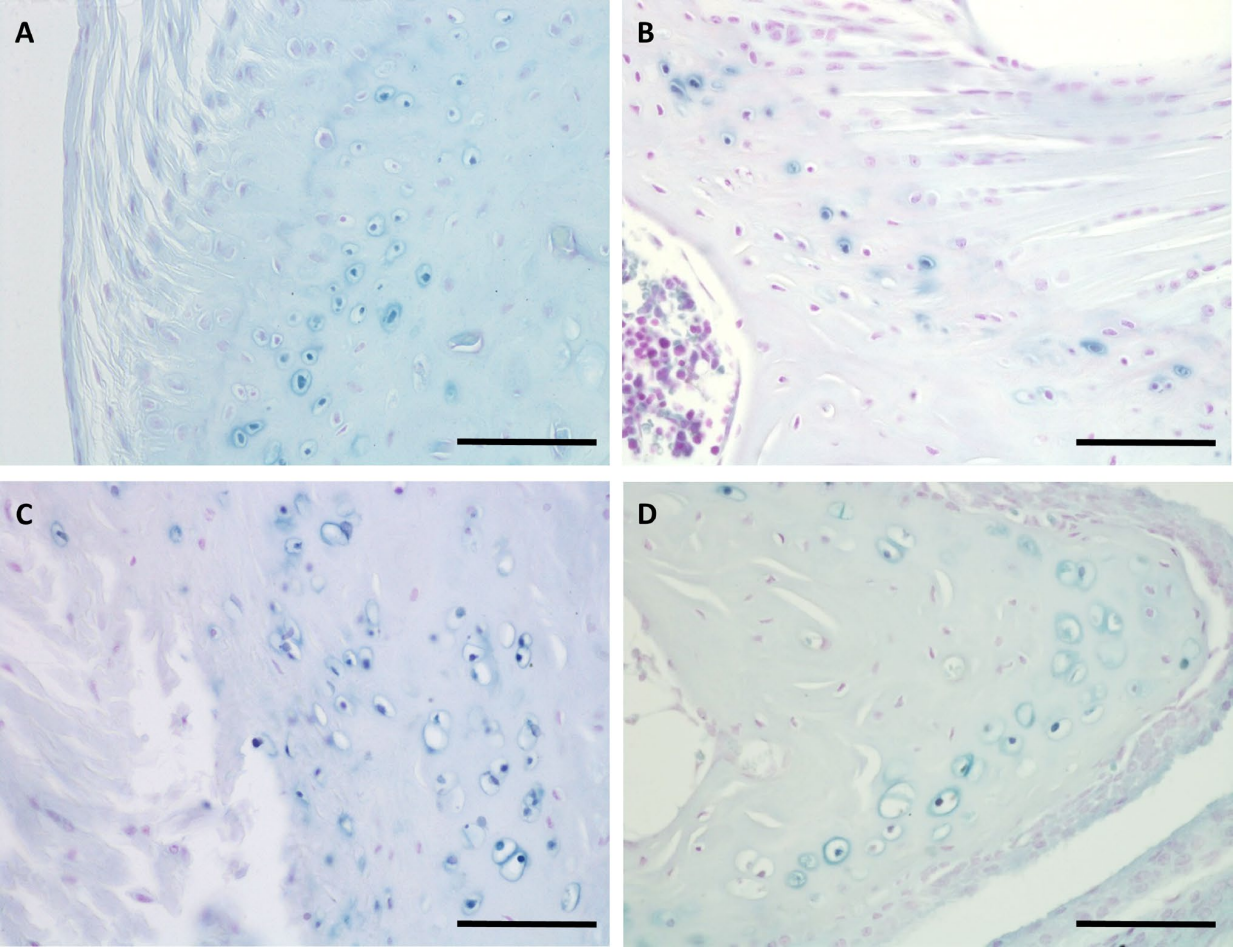
Presence of pigmented chondrons within the calcified fibrocartilage of entheses and the knee joint meniscus. **a** Clustering of pigmented chondrons adjacent to the insertion of the Achilles tendon on the calcaneus of the ankle. **b** Pigmented chondrons adjacent to the insertion of a cruciate ligament on the intercondylar area of the tibial plateau. **c** Pigmented chondrons observed in the knee joint collateral ligament entheses attached to the femoral condyles. **d** Pigmented chondrons within the calcified fibrocartilage of the meniscus of the knee joint. A from 65.7-week-old (F) BALB/c *Hgd*^*−/−*^. B from 40-week-old (M) *Hgd tm1a*^*−/−*^. C from 61.3-week-old (F) BALB/c *Hgd*^*−/−*^. D from 60-week-old (F) BALB/c *Hgd*^*−/−*^. Scale bar = 50 µm

Further to the observation that pigmentation increases linearly with age in the knee joint, the severity of pigmentation also appeared to be related to mechanical loading and force. This is best highlighted by the observation that significantly more pigmented chondrons (mean ± SEM) were observed within the endplates of the vertebral body of the 2nd lumbar (L2) IVD (48.4 ± 4.5) than in the endplates of an IVD from the base of the tail (2.2 ± 0.6) (*p* = 0.002) (Fig. 6a–e). Clustering of pigmented chondrons was observed in areas of greater tensile stress such as the insertion of tendons and ligaments in joints; for example, the insertion of the Achilles tendon on the calcaneus in the ankle (Fig. 7a), of the cruciate ligaments on the intercondylar area of the tibial plateau (Fig. 7b), and in the calcified fibrocartilage found within the entheses attached to the femoral condyle (Fig. 7c). Pigmentation was also observed within the calcified fibrocartilaginous meniscus of the knee (Fig. 7d).

In addition to musculoskeletal tissues, sections from the ear, tracheal and laryngeal cartilage, eye and heart of 52-week-old *Hgd tm1a*^*−/−*^ mice were stained and compared to 52-week-old *Hgd tm1a*^*−/*+^ (non-AKU) control tissues (all tissues from female mice except tracheal/laryngeal cartilage from males). Ochronotic pigmentation was not observed in the eye, the elastic cartilage of the pinna of the ear or the hyaline tracheal and laryngeal cartilages, and was comparable to the non-AKU control. In the heart, no ochronotic pigmentation was observed in the roots and walls of vessels or in the muscular wall/septum of the heart chambers.

## Discussion

Investigating the natural history of ochronotic pigmentation in AKU mice reveals which tissues are most susceptible to ochronosis and suggests links to physiological factors that may affect the severity of pigmentation. This information may give an insight into the mechanism of pigment formation/deposition. But perhaps more importantly, the location and pattern of ochronosis in AKU which is a rare form of osteoarthritis (OA) may reveal important changes that occur more generally in cartilage during ageing.

It is not currently known when and where ochronotic pigmentation in human AKU is first deposited in the body, due to the inaccessibility of joint tissues, with current assessments relying on externally visible pigmentation within the eye and ear cartilage [21, 30]. The initial site of pigment deposition in AKU mice, however, is clearly associated with chondrons within calcified cartilage, and this may also be the case in human AKU at a very young age. It is possible that pigment deposition is a time dependent process that takes many years to accumulate and become visible macroscopically, in addition to affecting tissues other than calcified cartilage, as is observed in end-stage human tissues after many decades of HGA exposure. If the lifespan of mice was longer, or if the disease process could somehow be accelerated, perhaps AKU mice would also eventually exhibit both macroscopic and widespread pigmentation of tissues including the hyaline cartilage and IVDs as seen in humans.

The progressive nature of ochronosis has previously been demonstrated in the ENU AKU mouse model; pigmentation in the BALB/c *Hgd*^*−/−*^ mouse was shown to begin at 15 weeks of age (mice aged 6, 8 and 15 weeks were investigated) and then to increase linearly with age in the knee joint ACC [15, 29]. Here we show that the progressive increase in the number of pigmented chondrons in the knee joint of BALB/c *Hgd*^*−/−*^ mice is both comparable and reproducible in the newer, targeted *Hgd tm1a*^*−/−*^ mouse model of AKU. Pigmented chondrons were observed here in *Hgd tm1a*^*−/−*^ mice at 7 weeks onwards, increasing in number with age. With almost identical ochronotic phenotypes, either model appears to be suitable for further research into ochronosis and can be used inter-changeably. To compare the progression of knee joint ochronosis of *Hgd tm1a*^*−/−*^ with the BALB/c *Hgd*^*−/−*^ model, the quantitation method previously described by Preston et al*.* [15] was used; however, we acknowledge that there are limitations with this method. The intensity of pigment staining within each chondron is not considered, and only one representative section from each joint is assessed. Assessing the intensity of pigmentation would require all sections to be stained at the same time which was not possible here. Future work could also consider assessing the mean number of pigmented chondrons over multiple sections from the joint. Despite these limitations, the scoring system here was adequate to show that pigmentation increases with age, and has also been used previously to demonstrate the effectiveness of a HGA-lowering drug at preventing chondron pigmentation in AKU mice [15, 29].

HGA is increased in the circulation from birth in AKU mice [28] and remains elevated to a similar degree across the lifetime [15, 29], therefore all tissues are exposed to HGA via extracellular fluid. Despite constant HGA exposure since birth, pigmented chondrons were not observed until approximately 10 weeks of age in mice, and other more vascularised connective tissues such as bone do not exhibit ochronosis. This time dependency for the onset of ochronosis and tissue specificity could suggest that changes in the ECM must occur to allow HGA/pigment deposition as described by the exposed collagen hypothesis [18], and that the matrix of ACC chondrons must undergo this unknown, pigment-favouring change prior to the matrix in other tissues such as in hyaline cartilage or tendons. Evidence from human tissues suggests that collagen is the binding site for HGA or HGA-derived pigment [13, 17, 19]. The exposed collagen hypothesis suggests that these binding sites over time become exposed due to both mechanical and chemical damage acquired through normal physiological loading and ageing, through loss of proteoglycans for example.

The observational data here in AKU mice support the exposed collagen hypothesis; pigmentation is associated with the collagen-rich matrix of calcified cartilage, pigmentation is increased with age and there appears to be an association between sites of greater mechanical loading and the location of pigmented chondrons. With normal cartilage ageing, chondrocyte function has been shown to decline, with decreased tissue turnover and renewal of the ECM, resulting in increased stiffness which alters the mechanical capacity of the cartilage, leading to increased susceptibility to destruction [31]. The early-onset and severe osteoarthritis that manifests in AKU is caused by the deposition of ochronotic pigment, which has been shown to make cartilage stiffer [5], accelerating the normal ageing process of the cartilage. A significantly greater number of pigmented chondrons was observed in the lumbar vertebral endplates, which would be expected to experience greater physiological compressive load, than in those found at the base of the tail. Axial compressive loading of the lumbar and tail (or caudal) discs in mice and rats has shown higher peak force in lumbar discs [32], in addition to an increased torsional modulus (with or without compressive load) observed in lumbar versus tail discs in rats [33]. Lumbar discs in mice are also stiffer than tail discs under compressive strain [34], highlighting the difference in normal physiological loading of these spinal regions. The entheses of ligaments and tendons are composed of both non-calcified and calcified fibrocartilage, that insert into subchondral bone, creating a gradient of increasing stiffness (transitioning from the ligament/tendon firstly to uncalcified fibrocartilage, then to calcified fibrocartilage and then bone) that can transfer tensile load from the ligament/tendon [35]. The high tensile strain of the anterior cruciate ligament at its insertion into the ACC of the medial tibia has been demonstrated ex vivo in an 8-week-old STR/Ort mouse (pre-OA) knee joint under loading conditions [36]. Clustering of pigmented chondrons was observed within the entheses of both ligaments and tendon in AKU mice, particularly the cruciate ligaments and Achilles tendon; ECM subjected to greater mechanical strain may therefore be more susceptible to pigmentation.

The association between physiological loading and pigmentation is further supported by observations in human tissues. Macroscopic pigmentation on the surface of human cartilage appears to be localised to areas of greater loading such as the medial tibial plateau of the knee, when compared to the lateral tibial plateau [5]. In the cardiovascular system the greater blood pressure of the systemic circulation appears to increase pigmentation, as the aortic and mitral heart valves were heavily pigmented while the tricuspid valve was unaffected and minimal pigmentation was observed in the pulmonary valve within the same individual [9]. Pigmentation has also been observed in the carotid sinus which is known to be exposed to more turbulent blood flow than the rest of the common carotid artery, which showed little pigmentation [37]. Lichtenstein and Kaplan also reported that pigment was barely found in veins, which are under less pressure than the arterial system [7]. Scleral pigmentation within the eye has also been postulated to be due to UV light damage and physical stress arising from ocular muscle contractions [16]. The pattern of pigmentation in both AKU mice and humans suggests that mechanical loading increases the susceptibly of ECM to pigmentation, as observed here in areas of greater compressive load and tensile strain in mice; however, this remains to be established by experimental intervention in mice.

In addition to tissue calcification and loading, differences in the composition of the ECM between tissues could also be a factor affecting ochronosis. Since collagen is the most abundant protein in the ECM, its distribution among different tissues and also its rate of turnover may explain why some tissues are more susceptible to ochronosis. If ochronosis is reliant on the deposition of HGA/HGA-derived pigment in a time-dependant manner, tissues that turnover more rapidly may prevent the accumulation of pigment by renewal of the ECM and its collagen component. Tissues with different ECM structural organisation also probably transmit force/load differently, and therefore damage to collagen for example produced by physical stress may occur at different rates. It is possible that the metabolic activity of cells may also have a role in pigmentation. In an in vitro cell model of ochronosis, it was shown culture media incubated with HGA took weeks to darken in the absence of cells, but only a few days in the presence of cells, suggesting that cellular metabolism may somehow accelerate pigment deposition [4]. Initial pigmentation in mice is associated with calcified cartilage chondrocytes (as both extra- and intracellular pigmentation) and their immediate pericellular matrix, and not the rest of the calcified cartilage matrix—perhaps the specific metabolic activity of calcified cartilage chondrocytes combined with the pericellular matrix creates an environmental niche that promotes pigmentation.

In general, the calcified layer of articular cartilage is a greatly understudied tissue compared to its neighbouring bone and hyaline cartilage. The initiation of pigmentation in AKU mice is associated with calcified cartilage, which in human AKU cartilage has then been shown to spread to the hyaline cartilage; this may reveal some important notions applicable to the ageing of cartilage, and some specific properties of the calcified cartilage. Firstly, the ACC must be permeable to small molecules such as HGA or HGA-derived structures to allow pigmentation. Secondly, if damage acquired through the natural ageing process and normal physiological loading causes the ACC to become susceptible to chemical attack, as illustrated by the deposition of HGA-related pigment in AKU, then it will also be susceptible to other small molecules that may lead to other detrimental changes to the structure and function of the ECM, that in turn may have a role in the progression of other age-related disease such as OA. The order of pigmentation in AKU would suggest that the ACC is the first of all joint connective tissues to become susceptible to attack. The ACC is the interface between the much stiffer underlying bone below and the less stiff hyaline cartilage above, acting as a transitional zone for the transmission of load. A nanoscale X-ray synchrotron study demonstrated that tissue compression within the medial tibia of an intact, loaded joint (ex vivo) was mostly observed in the ACC region, with some transmission into the SCB [36]. This high level of mechanical strain observed in the ACC may damage ECM components such as collagen, before other regions such as the hyaline cartilage above which may take longer to accumulate sufficient damage. The absence of pigmentation in the bone of AKU tissues suggest that the more highly calcified, and therefore more rigid bone matrix may protect the collagen from damage in bone so that it does not become susceptible to pigmentation. Changes to the ECM of the ACC appear to occur early in AKU mice allowing pigmentation, so it may be possible that these potential ECM changes in the ACC also occur in normal ageing that predisposes to conditions such as OA. The intrastrand disruption of the collagen triple helix by alterations in hydrogen bonding reported in pigmented AKU cartilage was also reported in non-AKU OA cartilage, suggesting a common change in the ECM that may be related to loading and ageing, predisposing to pigmentation and OA pathology [24].

No pigmentation was identified in the eyes, ears, tracheal/laryngeal cartilages, IVDs or the heart valves of the AKU mice, which are all sites of pigmentation in humans. Both BALB/c *Hgd*^*−/−*^ and *Hgd tm1a*^*−/−*^ mice exhibit the characteristic metabolic traits of AKU with increased HGA in the plasma and urine [15, 28, 29], but these models appear to represent only the early stage of ochronosis in humans. The initial site of ochronosis in human tissues may also be the chondrons of calcified cartilage, with other tissues subsequently becoming pigmented. We suggest that the deposition of ochronotic pigment is dependent on age-related changes in the organisation and/or composition of the ECM and may be accelerated by loading/physical stress. The focal nature of initial ochronotic pigmentation in AKU to the calcified cartilage may reveal the order of ECM damage that can lead to increased susceptibility to chemical attack and aberrant loading of cartilage, which could be a common mechanism in other age-related conditions such as OA.
